# Supplementary material for: HIVprotI: an integrated web based platform for prediction and design of HIV proteins inhibitors
Source: J Cheminform. 2018 Mar 9;10:12. doi: 10.1186/s13321-018-0266-y (PMC5845081; doi:10.1186/s13321-018-0266-y)
Supplement: Supplementary file 2 — Additional file 2. Source code of HIVProtI web server. [file 13321_2018_266_MOESM2_ESM.zip › HIVprotI_Source-code/hivprotI/weka-3-6-6/doc/weka/core/pmml/PMMLUtils.html]

PMMLUtils


---


|  |  |  |
| --- | --- | --- |
| **Overview** \| **Package** \| **Class** \| **Tree** \| **Deprecated** \| **Index** \| **Help** \| **Weka's home** | |  |
| **PREV CLASS**   **NEXT CLASS** | **FRAMES**    **NO FRAMES**     **All Classes** |  |
| SUMMARY: NESTED \| FIELD \| CONSTR \| METHOD | DETAIL: FIELD \| CONSTR \| METHOD |  |


---


## weka.core.pmml Class PMMLUtils

```
java.lang.Object
  weka.core.pmml.PMMLUtils
```

---

``` public class PMMLUtils extends java.lang.Object ```

Utility routines.

**Version:**
:   $Revision: 5562 $

**Author:**
:   Mark Hall (mhall{[at]}pentaho{[dot]}com)

---

**Constructor Summary**

`PMMLUtils()`


| **Method Summary** | |
| --- | --- |
| `static java.lang.String` | `pad(java.lang.String source, java.lang.String padChar, int length, boolean leftPad)`             Utility method to left or right pad strings with arbitrary characters. |

**Methods inherited from class java.lang.Object**

`equals, getClass, hashCode, notify, notifyAll, toString, wait, wait, wait`

**Constructor Detail**

### PMMLUtils

```
public PMMLUtils()
```


**Method Detail**

### pad

```
public static java.lang.String pad(java.lang.String source,
                                   java.lang.String padChar,
                                   int length,
                                   boolean leftPad)
```

:   Utility method to left or right pad strings with arbitrary characters.

    :   **Parameters:**: `source` - the source string: `padChar` - the character to pad with: `length` - the length of the resulting string: `leftPad` - pad to the left instead of the right **Returns:**: a padded string


---


|  |  |  |
| --- | --- | --- |
| **Overview** \| **Package** \| **Class** \| **Tree** \| **Deprecated** \| **Index** \| **Help** \| **Weka's home** | |  |
| **PREV CLASS**   **NEXT CLASS** | **FRAMES**    **NO FRAMES**     **All Classes** |  |
| SUMMARY: NESTED \| FIELD \| CONSTR \| METHOD | DETAIL: FIELD \| CONSTR \| METHOD |  |


---
